# Admission Liver Injury Is an Independent Predictor of Early and Overall Mortality in Hospitalized Patients with Moderate-to-Critical COVID-19: A Retrospective Cohort Study

**DOI:** 10.3390/life16091548

**Published:** 2026-09-16

**Authors:** Delia-Ionela Negru-Vodă, Ana Stemate, Lucian Negreanu

**Affiliations:** 1 Faculty of General Medicine, “Carol Davila” University of Medicine and Pharmacy, Dionisie Lupu Street, No. 37, Sector 2, 020021 Bucharest, Romania; lucian.negreanu@umfcd.ro; 2 Department of Gastroenterology 1, Emergency University Hospital, Splaiul Independentei Street, No. 169, Sector 5, 050098 Bucharest, Romania

**Keywords:** COVID-19, SARS-CoV-2, liver function tests, liver diseases, mortality, prognosis

## Abstract

**Background**: Liver injury (LI) is frequently observed in patients with COVID-19, particularly in severe forms of disease. However, its prognostic significance remains incompletely defined. **Aim**: To evaluate the association between LI at admission and clinical outcomes in hospitalized patients with moderate, severe, and critical COVID-19. **Methods**: This single-centre retrospective cohort study included 816 adults hospitalized with moderate-to-critical COVID-19 at a tertiary care hospital in Romania. LI was defined as elevations ≥ 2× the upper limit of normal (ULN) in alanine aminotransferase (ALT), aspartate aminotransferase (AST), alkaline phosphatase (ALP), gamma-glutamyl transferase (GGT), or total bilirubin (TB) at admission. Clinical, laboratory, imaging, and outcome data, including intensive care unit (ICU) admission, invasive mechanical ventilation (IMV), and mortality, were analysed. **Results**: LI was present in 34.9% of patients. Compared with patients without LI, affected patients more frequently exhibited inflammatory syndrome (98.7% vs. 95.2%, *p* = 0.017), elevated interleukin-6 (IL-6) levels (65.3% vs. 44.3%, *p* = 0.007), and extensive pulmonary involvement (>75% on chest computed tomography; 38.2% vs. 27.3%, *p* = 0.003). They were also more likely to require ICU admission (44.2% vs. 36.2%, *p* = 0.025) and IMV (30.9% vs. 23.5%, *p* = 0.011). Seven-day mortality (25.9% vs. 16.1%, *p* = 0.001) and overall mortality (49.8% vs. 36.8%, *p* < 0.001) were significantly higher among patients with LI. In multivariable analysis, LI remained independently associated with both 7-day and overall mortality. The association between admission LI and mortality remained consistent across pandemic waves, with no significant interaction between liver injury and wave (*p* = 0.731). **Conclusions**: LI at admission may help identify patients with COVID-19 at increased risk of adverse outcomes and represents a simple and widely available tool for early risk stratification and clinical management.

## 1. Introduction

The coronavirus disease 2019 (COVID-19) pandemic, caused by severe acute respiratory syndrome coronavirus 2 (SARS-CoV-2), resulted in more than 7 million deaths worldwide, representing an unprecedented global health crisis [1]. Regardless of the viral variants that emerged throughout the course of the pandemic, COVID-19 represented not only a major public health burden but also a global economic, social, and educational challenge, profoundly impacting and reshaping contemporary society [2].

The clinical presentation of COVID-19 is highly heterogeneous, ranging from asymptomatic infection to life-threatening conditions such as acute respiratory distress syndrome (ARDS) and multiple organ failure (MOF) [3].

Beyond pulmonary involvement, SARS-CoV-2 may disseminate systemically due to the widespread distribution of angiotensin-converting enzyme 2 (ACE2), the principal receptor mediating viral entry into host cells. The liver is considered one of the most frequently affected extrapulmonary organs, with liver injury (LI) reported in 19–76% of patients diagnosed with COVID-19 [4]. In severe forms of disease, the prevalence of LI may reach up to 93%, suggesting a potential association between hepatic involvement and mortality [5]. The mechanisms underlying LI in COVID-19 are complex and remain incompletely understood. Current evidence suggests a multifactorial pathogenesis involving direct viral cytotoxicity, exaggerated immune response accompanied by cytokine storm, hypoxia-reperfusion imbalance, endothelial dysfunction, microthrombosis, disruption of the gut–liver axis and intestinal microbiota, as well as drug-induced hepatotoxicity [6,7].

LI in COVID-19 has been frequently associated with male sex, older age, chronic liver disease, systemic proinflammatory status, and exposure to potentially hepatotoxic medications [8]. Elevated aminotransferase levels, hypoalbuminemia, and abnormalities in total bilirubin (TB), alkaline phosphatase (ALP), and gamma-glutamyl transferase (GGT) have been associated with severe disease, intensive care unit (ICU) admission, and mortality. Given the close association between liver biochemical abnormalities and severe COVID-19, understanding this relationship may facilitate early identification of high-risk patients requiring intensive care and contribute to optimizing therapeutic management [9].

Beyond the acute phase of COVID-19, persistent liver biochemical abnormalities have also been reported after recovery. Previous studies have described persistent elevations of aminotransferases and cholestatic markers during post-COVID follow-up, particularly among patients with hepatic involvement during the acute phase [10]. These observations have raised interest in the potential long-term hepatic consequences of SARS-CoV-2 infection and in the role of post-discharge liver biochemical monitoring in selected patients [11].

This study aimed to evaluate the clinical and laboratory characteristics of hospitalized patients with moderate-to-critical COVID-19 and to assess the prognostic value of liver injury at admission for adverse clinical outcomes and mortality.

## 2. Materials and Methods

During the COVID-19 pandemic, major healthcare reorganization resulted in the conversion of several hospital departments into dedicated COVID-19 units. In this setting, our department functioned as a referral unit for hospitalized patients with moderate-to-critical COVID-19.

Considering the significant hepatic involvement associated with SARS-CoV-2 infection, we aimed to present our experience from the perspective of a gastroenterology department through a retrospective study including 816 patients with moderate, severe, or critical COVID-19 admitted between 1 January 2020 and 30 November 2021.

For the analysis according to pandemic waves, patients were grouped based on the calendar period of hospital admission, with admission occurring between two consecutive waves assigned to the temporally closest wave. These groups represent epidemiological periods rather than individually confirmed SARS-CoV-2 variants, as viral sequencing data were not available. The small number of patients included during the first wave reflects the organization of COVID-19 care in Romania during the early phase of the pandemic, when patients were primarily managed in designated infectious diseases hospitals; as hospital demand increased, COVID-19 care was progressively extended to other hospital units, including our center. Patients were followed until discharge, death, or transfer to another healthcare facility.

Study population. The *inclusion criteria* were: age ≥ 18 years, signed informed consent for participation in clinical studies, SARS-CoV-2 infection confirmed by polymerase chain reaction (PCR), and liver biochemical tests assessed at admission. *The exclusion criteria* were: asymptomatic patients with positive SARS-CoV-2 PCR, patients admitted for other conditions who subsequently developed SARS-CoV-2 infection during hospitalization, pregnant women, patients with insufficient clinical data, patients with chronic liver disease, patients unable to consent due to impaired mental status.

Written informed consent was obtained from all patients included in the study. The study was approved by the Ethics Committee of the Bucharest Emergency University Hospital (32746/2026).

The variables analyzed during the study included demographic characteristics, comorbidities, clinical presentation, laboratory findings at admission, imaging features and clinical outcomes. Laboratory assessment included serum levels of aspartate aminotransferase (AST), alanine aminotransferase (ALT), ALP, GGT, TB, fibrinogen, C-reactive protein (CRP), ferritin, interleukin-6 (IL-6), as well as white blood cells, platelet, neutrophil, and lymphocyte counts. Imaging evaluation consisted of chest computed tomography (CT), with or without contrast enhancement, used to assess the extent of pulmonary involvement. Clinical outcomes included ICU admission, ICU length of stay, requirement for invasive mechanical ventilation (IMV) or non-invasive mechanical ventilation (NIMV), total hospital length of stay, 7-day mortality, overall mortality and transfer to another medical department.

LI was defined as elevation of at least one of the following parameters ≥ 2× the upper limit of normal (ULN): ALT, AST, ALP, GGT, or TB. Given the lack of a universally accepted definition of liver injury in COVID-19, the ≥2× ULN cutoff was selected to identify relevant liver biochemical abnormalities while excluding minor elevations. Based on the presence of LI at admission, patients were stratified into two study groups: patients without LI (Group 1) and patients with LI (Group 2). According to the biochemical pattern of hepatic involvement, LI was further classified as hepatocellular (AST and/or ALT ≥ 2× ULN), cholestatic (ALP and/or GGT and/or TB ≥ 2× ULN), or mixed, defined by the simultaneous elevation of hepatocellular and cholestatic markers [12].

Clinical data were retrieved from electronic medical records and systematically recorded in a dedicated study database. Statistical analysis was performed using IBM SPSS Statistics for Windows, version 20.0 (IBM Corp., Armonk, NY, USA). Continuous variables were summarized as mean ± standard deviation (SD) or median and interquartile range (IQR), according to their distribution, while categorical variables were expressed as absolute frequencies and percentages. The independent-samples *t*-test and Mann–Whitney U test were used for between-group comparisons of continuous variables, as appropriate. Categorical variables were compared using the chi-square test. For ordinal CRP categories, the Linear-by-Linear Association test was additionally used to assess the presence of a trend across increasing CRP categories. Comparisons among the three patterns of LI (hepatocellular, cholestatic, and mixed) were performed using the Kruskal–Wallis test for continuous variables. When a significant overall difference was identified, pairwise comparisons were performed using the Mann–Whitney U test with Bonferroni correction. Categorical variables were compared using the chi-square test or the Fisher–Freeman–Halton exact test, as appropriate. Multivariable binary logistic regression models were constructed to assess the independent association of LI at admission with overall mortality, 7-day mortality, and ICU admission. Results are reported as adjusted odds ratios (ORs) with 95% confidence intervals (CIs). Only patients with available outcome data and complete data for the variables included in each model were analyzed. Missing data were handled using available-case analysis for descriptive and comparative analyses and complete-case analysis for multivariable regression; no imputation of missing values was performed. An additional analysis was performed according to pandemic wave. Differences in the association between LI and mortality across pandemic waves were assessed by including an interaction term between LI and pandemic wave. Pandemic wave was also included in the multivariable mortality model to determine whether the association between LI and mortality remained independent of the pandemic period. Due to the small number of patients with known outcomes during the first wave, wave-specific mortality analyses were restricted to waves 2–4. All statistical tests were two-sided, and a *p*-value < 0.05 was considered statistically significant.

## 3. Results

### 3.1. Characteristics of the Study Population

A total of 816 patients were included in the study, with a mean age of 68.93 ± 13.63 years; 427 (52.3%) were male and 389 (47.7%) were female. Most patients originated from urban areas (80.3%) and were predominantly unvaccinated (94.1%). Among vaccinated individuals, 1.3% had received one vaccine dose and 4.5% had received two doses. The most frequent comorbidity was cardiovascular disease, identified in 77.6% of patients, followed by diabetes mellitus (35.9%), obesity (19.1%), pulmonary disease (12.4%), metabolic syndrome (9.9%), and malignancies (9.6%). The mean interval between symptom onset and presentation to the Emergency Department was 6.15 ± 3.48 days. Respiratory symptoms (rhinorrhea, nasal congestion, anosmia, cough, dyspnea, and tachypnea) were reported in 87.6% of cases, fever and chills in 52.5% and gastrointestinal symptoms (nausea, vomiting, ageusia, loss of appetite, abdominal pain, and diarrhea) in 18.4% of patients. At admission, leukocytosis was identified in 38.8% of patients, lymphopenia in 72.3%, neutrophilia in 41.3%, and thrombocytosis in 8.1%. The median (interquartile range (IQR)) neutrophil-to-lymphocyte ratio (NLR) was 9.25 (4.71; 16.78), while the platelet-to-lymphocyte ratio (PLR) was 320 (188.29; 478.75). Since different assays were used for CRP determination, CRP levels were categorized as <5× ULN, 5–10× ULN, and >10× ULN. Accordingly, 23.4% of patients were included in the first category, 16.9% in the second, while the majority (59.7%) presented CRP levels exceeding 10× ULN at admission. An additional variable, defined as “inflammatory syndrome,” included patients with elevated CRP according to the criteria above and/or elevated fibrinogen (>498 mg/dL) or ferritin levels (>150 ng/mL in women and >300 ng/mL in men). Based on these criteria, inflammatory syndrome was identified in 672/697 patients (96.4%) at admission. Pulmonary involvement was assessed using chest CT, with or without contrast enhancement when indicated. Most patients (40.4%) presented pulmonary involvement affecting 25–75% of both lung fields. A total of 109 patients (13.3%) were transferred to other medical departments, while 318 of 816 patients (39.0%) required ICU admission. The median (IQR) ICU length of stay was 6 (2; 11.25) days. During hospitalization, 103 patients (12.6%) required NIMV, 213 (26.1%) required IMV, while 500 patients (61.3%) did not require ventilatory support. The median (IQR) duration of hospitalization was 10 (7; 15) days after exclusion of transferred patients, in order to avoid artificial shortening of hospitalization duration due to early transfer following admission. Among the 707 patients with known final outcomes, 293 died during hospitalization (41.4%). Seven-day mortality data were available for 714 patients, of whom 140 died within the first 7 days after admission (19.6%). Patients transferred to other medical units without available post-transfer follow-up were excluded from the corresponding mortality analyses.

### 3.2. Comparative Analysis of Patients Without LI at Admission (Group 1) vs. Patients with LI at Admission (Group 2): Clinical and Paraclinical Characteristics at Hospital Admission

In order to assess the prognostic significance of LI at admission on disease progression and mortality, a comparative analysis between Group 1 and Group 2 was performed (Table 1). Among the 816 patients included in the study, 531 (65.1%) had no evidence of LI at admission, whereas 285 patients (34.9%) presented abnormal liver biochemical parameters. AST was the most frequently altered parameter, identified in 223/285 patients (78.2%), followed by ALT in 109 patients (38.2%), GGT in 83 (29.1%), and ALP in 42 (14.7%), while elevated TB levels were observed in only 30 patients (10.5%) from Group 2. Demographic analysis showed no statistically significant differences between the two groups regarding sex distribution, area of residence, or vaccination status. However, patients from Group 1 were significantly older compared with those from Group 2 (69.75 ± 13.2 years vs. 67.40 ± 14.29 years, *p* = 0.019). Regarding comorbidities, pre-existing pulmonary disease and diabetes mellitus were significantly more prevalent in Group 1 than in Group 2 (14.3% vs. 8.8%, *p* = 0.022; 39.2% vs. 29.8%, *p* = 0.008, respectively), whereas obesity was significantly more frequent among patients with LI (23.2% vs. 16.9%, *p* = 0.032). No significant differences were observed between the two groups with respect to clinical presentation at admission, respiratory manifestations remaining the predominant symptom category in more than 80% of patients in both groups. Owing to the retrospective design of the study, laboratory parameters were analyzed in a variable number of patients depending on data availability; the number of evaluated cases for each parameter is presented in Table 1. A significantly higher prevalence of inflammatory syndrome was identified in Group 2 compared with Group 1 (98.7% vs. 95.2%, *p* = 0.017), together with increased IL-6 levels (65.3% vs. 44.3%, *p* = 0.007). The association between CRP levels and LI was assessed using the chi-square test for independence. Although the overall association did not reach statistical significance (χ^2^(2) = 5.501, *p* = 0.064), the Linear-by-Linear Association test demonstrated a significant ordinal trend (χ^2^(1) = 4.101, *p* = 0.043). No statistically significant differences were identified regarding leukocytosis, lymphopenia, neutrophilia, thrombocytosis, NLR, or PLR. Patients with LI at admission more frequently presented extensive pulmonary involvement (>75%) compared with patients without LI (38.2% vs. 27.3%, *p* = 0.003).

The number of available observations varied for some laboratory parameters due to missing data. Percentages were calculated based on available data. Continuous variables were compared using the independent-samples *t*-test or Mann–Whitney U test, as appropriate. Categorical variables were compared using the chi-square test.

### 3.3. Comparative Analysis of Clinical Course and Prognosis According to the Presence of LI at Admission

**Figure 1:**
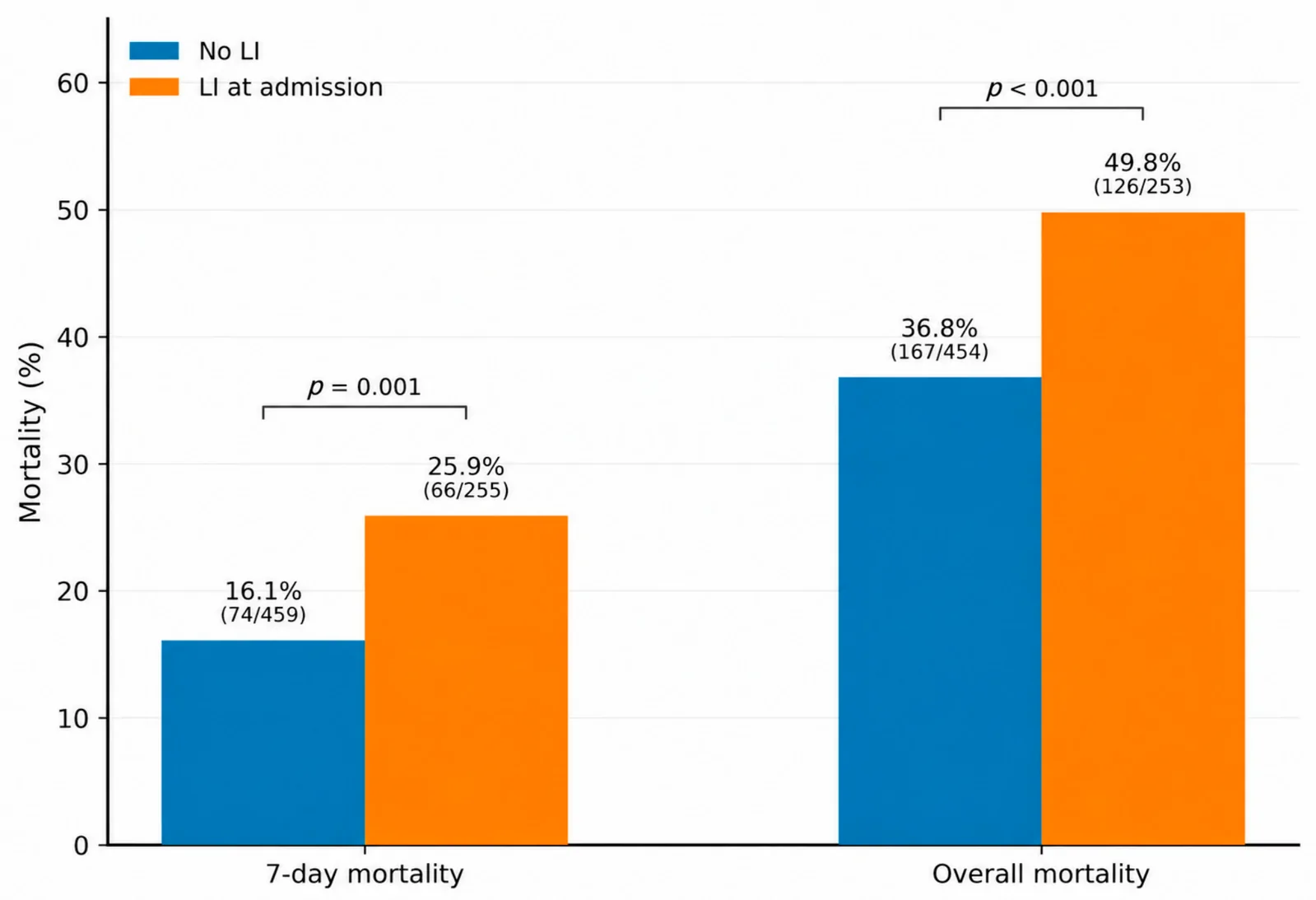
Mortality according to liver injury status at admission. Seven-day and overall mortality were significantly higher in patients with liver injury at admission compared with patients without liver injury (25.9% vs. 16.1%, *p* = 0.001 and 49.8% vs. 36.8%, *p* < 0.001 respectively).

The analysis of clinical outcomes (Table 2) showed no statistically significant differences between the two groups in terms of overall hospital length of stay (10.50 (7–15) vs. 10 (6–15) days, *p* = 0.250) or ICU stay duration (6 (3–12) vs. 5 (2–11) days, *p* = 0.141). Nevertheless, ICU admission was required significantly more frequently in Group 2 than in Group 1 (44.2% vs. 36.2%, *p* = 0.025). Similarly, the requirement for invasive mechanical ventilation was significantly higher among patients in Group 2 (30.9% vs. 23.5%, *p* = 0.011). One of the most important findings of the study was the association between LI at admission and mortality (Figure 1). Both 7-day mortality (25.9% vs. 16.1%, *p* = 0.001) and overall mortality were significantly higher among patients in Group 2 compared to those in Group 1 (49.8% vs. 36.8%, *p* < 0.001).

Mortality percentages were calculated based on patients with available outcome data. Continuous variables were compared using the Mann–Whitney U test, and categorical variables using the chi-square test.

### 3.4. Comparative Analysis According to the Pattern of LI at Admission

To assess whether the pattern of LI at admission influenced clinical evolution or had prognostic value regarding mortality, patients in Group 2 were further classified according to the type of LI as follows: hepatocellular injury (AST and/or ALT ≥ 2× ULN), cholestatic injury (ALP and/or GGT and/or total bilirubin ≥ 2× ULN), and mixed injury (patients presenting both hepatocellular and cholestatic abnormalities). Among the 285 patients with LI, 191 had a hepatocellular pattern, 39 a cholestatic pattern, and 55 a mixed pattern of LI (Table 3). Comparative analysis showed no statistically significant differences regarding sex distribution, most comorbidities, symptom profile, imaging findings, ICU admission, need for mechanical ventilation, or overall in-hospital mortality. Patients with mixed LI were significantly younger than those with hepatocellular injury (Mann–Whitney U test with Bonferroni correction), with median ages of 64 (52–70) years versus 70 (62–80) years, respectively (*p* < 0.001). No significant differences were identified between the cholestatic and hepatocellular groups or between the cholestatic and mixed-pattern groups after adjustment for multiple comparisons. Cardiovascular comorbidities were significantly more frequent in patients with mixed LI compared with the other groups (*p* = 0.024). NLR values did not differ significantly among the three groups (*p* = 0.576), whereas PLR values showed a borderline statistically significant difference (*p* = 0.050), with a trend toward higher values in the hepatocellular injury group. Due to the small sample size in some subgroups, comparative analysis of inflammatory syndrome was performed using the Fisher–Freeman–Halton test. Inflammatory syndrome was significantly more frequent in patients with hepatocellular and mixed LI compared with those with cholestatic injury (*p* = 0.026). Patients with cholestatic LI had the longest hospital stay, with a median duration of 13 (7.5–22) days, compared with 9.5 (5–14) days in the hepatocellular group and 11 (7–15) days in the mixed-pattern group (*p* = 0.035). Seven-day mortality differed significantly among the three LI patterns (*p* = 0.039), whereas overall in-hospital mortality did not (*p* = 0.361).

An additional analysis was performed according to pandemic waves. Of the 816 patients, 12 were included in the first wave, 213 in the second, 267 in the third, and 324 in the fourth wave. Due to the small number of patients with known outcomes during the first wave, wave-specific mortality analyses were restricted to waves 2–4. The association between LI and mortality showed the same direction across these waves, with no significant interaction between LI and pandemic wave (*p* = 0.731). After adjustment for pandemic wave, LI remained independently associated with mortality (OR = 1.639, 95% CI 1.042–2.578, *p* = 0.033).

**Figure 2:**
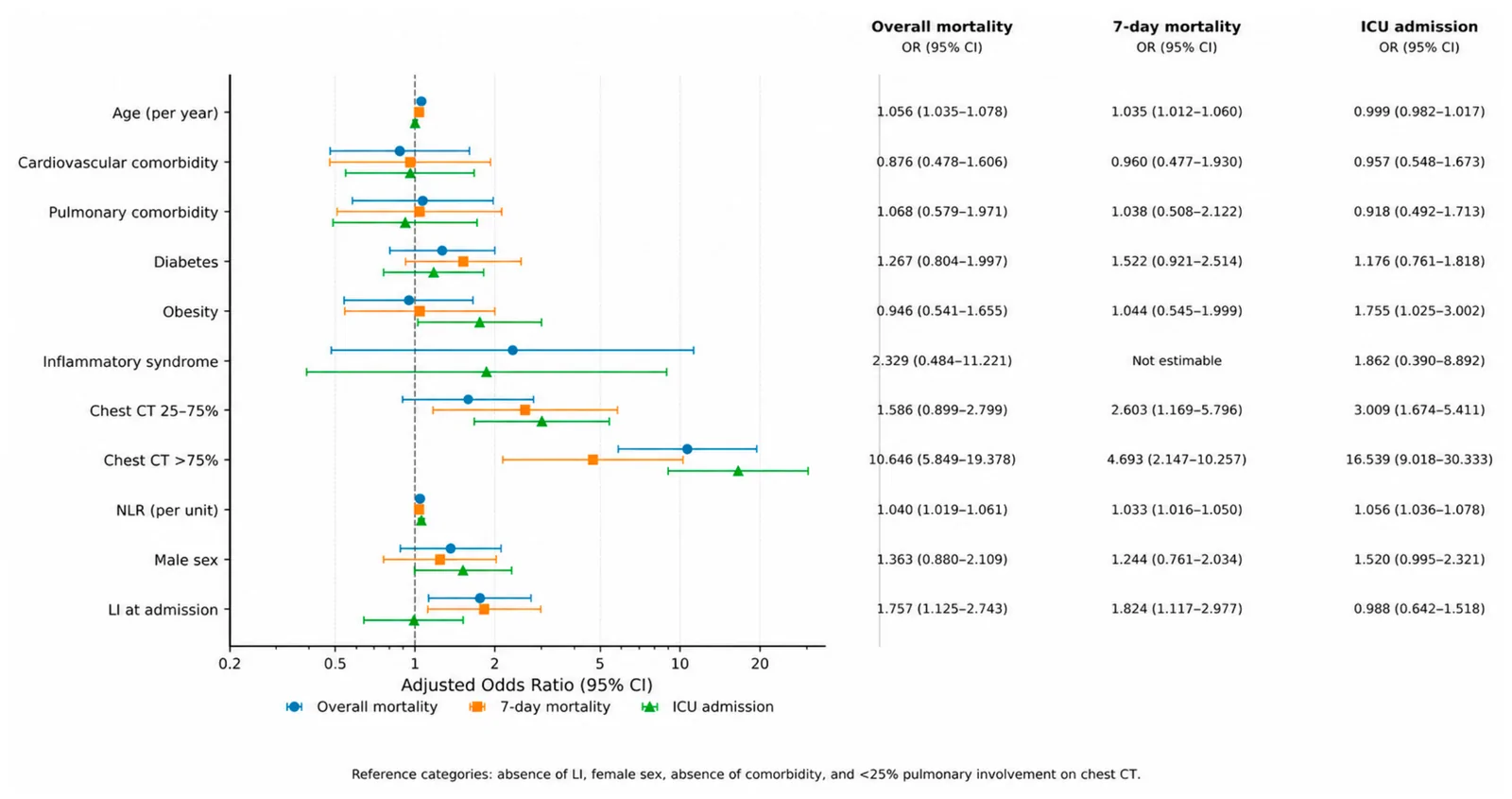
Forest plot of the multivariable logistic regression analyses for overall mortality, 7-day mortality, and ICU admission. Adjusted odds ratios (ORs) and 95% confidence intervals (CIs) are shown for each variable included in the models. The vertical dashed line indicates an OR of 1. Reference categories were absence of LI, female sex, absence of comorbidity, and <25% pulmonary involvement on chest CT. LI, liver injury; NLR, neutrophil-to-lymphocyte ratio; ICU, intensive care unit.

To evaluate the independent association of LI with patient outcomes, multivariable binary logistic regression models were constructed adjusting for the main clinical and paraclinical factors potentially associated with disease severity (Table 4). The logistic regression models were statistically significant for all three analyzed outcomes. LI at admission remained independently associated with overall mortality (OR = 1.757, 95% CI 1.125–2.743, *p* = 0.013) and 7-day mortality (OR = 1.824, 95% CI 1.117–2.977, *p* = 0.016), but not with ICU admission (OR = 0.988, 95% CI 0.642–1.518, *p* = 0.955). Age was independently associated with both overall and 7-day mortality, with each additional year corresponding to higher odds of death (OR = 1.056, *p* < 0.001 and OR = 1.035, *p* = 0.003, respectively). Higher NLR values were independently associated with all three analyzed outcomes. The extent of pulmonary involvement on chest CT was strongly associated with adverse outcomes, with >75% lung involvement showing the largest effect estimates for overall mortality (OR = 10.646, 95% CI 5.849–19.378, *p* < 0.001), 7-day mortality (OR = 4.693, 95% CI 2.147–10.257, *p* < 0.001), and ICU admission (OR = 16.539, 95% CI 9.018–30.333, *p* < 0.001), compared with <25% lung involvement. Among the other clinical variables, obesity was independently associated with ICU admission (OR = 1.755, 95% CI 1.025–3.002, *p* = 0.040), while male sex showed a trend toward statistical significance (OR = 1.520, 95% CI 0.995–2.321, *p* = 0.053). Overall, these findings indicate that LI at admission was independently associated with higher odds of both overall and 7-day mortality after adjustment for major clinical and paraclinical markers of disease severity. The adjusted associations of the variables included in the multivariable models with overall mortality, 7-day mortality, and ICU admission are graphically presented in Figure 2.

## 4. Discussion

Our single-center study, conducted in a cohort of 816 patients with moderate, severe, and critical COVID-19 hospitalized at a tertiary referral hospital in Romania, identified a prevalence of LI at admission of 34.9%, consistent with data reported in the recent literature [7,8]. Previous studies have reported abnormalities in liver biochemical tests in approximately 20–45% of patients with COVID-19, particularly among those with moderate and severe disease [7,8]. Patients presenting with LI at admission were significantly younger than those without LI, although the absolute difference in mean age between the two groups was modest. While this finding may appear counterintuitive, given the generally greater vulnerability of older patients to organ injury, age alone may not fully account for the occurrence of liver biochemical abnormalities in COVID-19. Notably, obesity was significantly more prevalent among patients with LI in our cohort, suggesting that metabolic risk factors may have contributed to hepatic susceptibility despite the slightly younger age of these patients. This observation should, however, be interpreted cautiously, as our data do not establish a causal relationship between obesity and LI in younger patients. Previous studies have similarly emphasized the contribution of obesity and metabolic dysfunction to COVID-19 severity and hepatic involvement [13,14]. No significant differences in clinical presentation were observed between the two groups, with respiratory symptoms representing the predominant manifestation in both cohorts. These findings suggest that LI is not associated with a distinct clinical phenotype at admission, an observation similarly reported in other cohorts of patients with COVID-19 [8].

Although no significant differences were identified regarding leukocytosis, lymphopenia, neutrophilia, thrombocytosis, NLR, or PLR, patients with LI more frequently exhibited inflammatory syndrome and elevated IL-6 levels. Moreover, CRP analysis demonstrated a significant ordinal association between increasing inflammatory marker levels and the presence of LI. These observations further support the central role of systemic inflammation in the pathogenesis of SARS-CoV-2-associated LI, a mechanism commonly mediated through cytokine storm, endothelial dysfunction, and hepatic microvascular impairment [15,16].

Patients with LI more frequently presented extensive pulmonary involvement and more often required ICU admission and IMV compared to patients without LI, suggesting a more severe clinical course. These findings are in agreement with current evidence describing liver biochemical abnormalities as markers of systemic disease severity and multiorgan involvement in severe COVID-19 [7,8]. Furthermore, LI at admission was associated with significantly higher rates of both early and overall mortality, supporting the unfavorable prognostic significance of abnormal liver biochemical parameters in patients with SARS-CoV-2 infection [9,17].

To assess the independent prognostic value of LI, multivariable models were adjusted for established clinical and paraclinical markers of COVID-19 severity. LI at admission remained independently associated with both overall and 7-day mortality, but not with ICU admission. The association between LI and mortality also remained significant after accounting for pandemic wave, suggesting that the prognostic value of admission LI was not explained by differences between pandemic periods. Importantly, these findings should not be interpreted as indicating that liver injury is a primary driver of COVID-19 severity. Rather, LI may represent one component of the multisystem response to severe infection and may provide prognostic information alongside other clinical, inflammatory, pulmonary, and endothelial markers. As expected, age, NLR, and the extent of pulmonary involvement were also associated with adverse outcomes, while obesity was independently associated with ICU admission, in accordance with previously reported predictors of severe COVID-19 [18,19,20]. Male sex showed only a trend toward association with ICU admission in our cohort, although it has previously been associated with severe disease progression and mortality [21]. Following the stratification of patients with LI into the three categories—hepatocellular, cholestatic, and mixed LI—the potential prognostic implications of the different patterns of LI were further evaluated. The analysis demonstrated a clear predominance of the hepatocellular pattern, identified in 67% of patients. Mixed LI was observed in 19.3% of cases, whereas the cholestatic pattern was identified in 13.7% of patients. These findings are consistent with the current literature, in which hepatocellular injury represents the most frequent form of liver involvement associated with SARS-CoV-2 infection [5,7,12]. The predominance of the hepatocellular pattern may be explained by mechanisms of hepatocellular injury related to systemic inflammation, hypoxia, and microvascular dysfunction, processes that more commonly lead to elevated transaminase levels than to the development of a cholestatic pattern [15,22]. Patients presenting with a mixed pattern of LI were significantly younger compared to those with hepatocellular injury. Data regarding age-related differences among the various patterns of LI in COVID-19 remain limited; however, existing evidence suggests that hepatocellular, cholestatic, and mixed patterns may reflect distinct underlying pathophysiological mechanisms. In our cohort, the younger age of patients with mixed LI may indicate a greater contribution of metabolic dysfunction and systemic inflammatory response, whereas hepatocellular injury in older patients may be more frequently related to hypoxia, biological frailty, and the systemic involvement associated with severe forms of the disease. In contrast to several international cohorts that reported a poorer prognosis associated with cholestatic and mixed patterns of LI, particularly regarding mortality and critical disease progression [23], no significant differences were identified in our cohort among the three patterns of LI with respect to pulmonary involvement, ICU admission, requirement for mechanical ventilation, or overall in-hospital mortality. However, 7-day mortality differed significantly among the three LI patterns (*p* = 0.039), with the highest rate observed in patients with hepatocellular injury. These discrepancies may reflect the heterogeneity of the studied populations, the relatively small size of the cholestatic and mixed subgroups, or differences in the underlying pathophysiological mechanisms implicated in COVID-19-associated LI. Patients with cholestatic LI exhibited the longest duration of hospitalization compared to the other patterns of LI. Similar findings have also been reported in other studies, in which the cholestatic pattern was associated with a more prolonged clinical course and delayed recovery [22,24]. A possible explanation may be related to cholangiocellular injury associated with SARS-CoV-2 infection, considering the increased expression of ACE2 receptors in cholangiocytes [24].

## 5. Limitations

Our study has several limitations that should be considered when interpreting the results. First, the retrospective design limited the availability of certain clinical and laboratory data. Biomarkers that could have helped distinguish hepatic from extrahepatic sources of AST elevation, such as creatine kinase (CK) and creatine kinase-MB (CK-MB), were not routinely available. This limitation is clinically relevant given that AST was the most frequently elevated biochemical marker in our cohort. As AST is not liver-specific, a potential contribution of skeletal muscle, cardiac, or endothelial injury to AST elevation, particularly in patients with severe COVID-19, cannot be excluded. Therefore, AST elevations should be interpreted within the broader biochemical and clinical context rather than as a liver-specific marker in isolation. Unfortunately, markers of hepatic synthetic and metabolic function, including total protein, albumin, cholesterol, urea, and butyrylcholinesterase, were not systematically available and could not be included in the analysis. Accordingly, the present study evaluates liver biochemical injury rather than comprehensive hepatic functional impairment. Specific markers of endothelial dysfunction, including von Willebrand factor, and ADAMTS-13, were not available in our cohort; therefore, the contribution of endothelial injury could not be specifically assessed. In addition, liver injury severity could not be further assessed using higher biochemical cutoffs, such as AST/ALT ≥ 5× ULN, as the numerical transaminase values required for this analysis were not available in the analytical dataset. Furthermore, complete data regarding medication exposure prior to hospital admission were not available for a specific assessment of its potential contribution to liver biochemical abnormalities; therefore, drug-induced liver injury as a potential confounding factor cannot be entirely excluded. In addition, the use of multiple laboratory assay kits with different cut-off values did not allow the analysis of certain parameters as continuous numerical variables, an aspect that might have improved the statistical accuracy and comparability of the results. The retrospective design may also have introduced selection bias. Furthermore, the single-center design of the study and the relatively limited number of patients may reduce the generalizability of the findings. Regarding anamnestic data, the inclusion of patients with moderate, severe, and critical forms of COVID-19 may have influenced the accuracy of certain information reported at admission, such as symptom duration prior to presentation. Although patients with known chronic liver disease were excluded, detailed information regarding alcohol consumption and pre-COVID liver biochemical values was not systematically available. Therefore, the possibility that some liver biochemical abnormalities detected at admission were pre-existing cannot be entirely excluded. Another important limitation is the lack of available follow-up data after the transfer of some patients to other medical centers, which prevented a complete assessment of mortality across the entire cohort. Furthermore, post-discharge follow-up data were not available; therefore, the present study cannot assess the persistence of liver biochemical abnormalities or post-COVID hepatobiliary dysfunction.

## 6. Conclusions

In conclusion, LI at admission was frequently encountered in patients with moderate, severe, and critical forms of COVID-19 and was associated with enhanced systemic inflammation, more extensive pulmonary involvement, and unfavorable clinical outcomes. Importantly, LI remained independently associated with both overall and 7-day mortality after adjustment for major clinical and paraclinical markers of disease severity, supporting its value as an independent prognostic indicator in hospitalized patients with COVID-19. The predominance of the hepatocellular pattern suggests a major contribution of systemic inflammation, hypoxia, and microvascular dysfunction to the pathogenesis of COVID-19-associated LI. Overall, the assessment of liver biochemical parameters at admission may provide valuable prognostic information and contribute to early risk stratification and clinical decision-making in patients hospitalized with COVID-19.

## Figures and Tables

**Table 1:** Baseline Clinical and Paraclinical Characteristics According to Liver Injury Status at Admission.

Variable	Group 1 (No LI)	Group 2 (LI at Admission)	*p*-Value
**Gender, n (%)**			0.294
Male	285 (53.7%)	142 (49.8%)	
Female	246 (46.3%)	143 (50.2%)	
**Age (years), Mean ± SD**	69.8 ± 13.2	67.4 ± 14.3	**0.019**
**Place of residence, n (%)**			0.152
Rural	97 (18.3%)	64 (22.5%)	
Urban	434 (81.7%)	221 (77.5%)	
**Vaccination status, n (%)**			0.237
Unvaccinated	499 (94.0%)	269 (94.4%)	
Vaccinated with 1 dose	5 (0.9%)	6 (2.1%)	
Vaccinated with 2 doses	27 (5.1%)	10 (3.5%)	
**Comorbidities, n (%)**			
Cardiovascular	417 (78.5%)	216 (75.8%)	0.371
Pulmonary	76 (14.3%)	25 (8.8%)	**0.022**
Malignancies	54 (10.2%)	24 (8.4%)	0.418
Diabetes	208 (39.2%)	85 (29.8%)	**0.008**
Obesity	90 (16.9%)	66 (23.2%)	**0.032**
Metabolic Syndrome	46 (8.7%)	35 (12.3%)	0.090
**Symptoms, n (%)**			
Fever	279 (52.5%)	149 (52.3%)	0.943
Digestive	101 (19.0%)	49 (17.2%)	0.520
Respiratory	458 (86.3%)	257 (90.2%)	0.105
**Laboratory parameters, n (%)**			
Leukocytosis (n = 694), n/N (%)	166/449 (37.0%)	103/245 (42.0%)	0.190
Lymphopenia (n = 664), n/N (%)	305/429 (71.1%)	175/235 (74.5%)	0.353
Neutrophilia (n = 664), n/N (%)	174/429 (40.6%)	100/235 (42.6%)	0.618
Thrombocytosis (n = 694), n/N (%)	31/450 (6.9%)	25/244 (10.2%)	0.121
CRP (n = 531), n/N (%)			0.064
<5× ULN	91/343 (26.5%)	33/188 (17.6%)	
5–10× ULN	55/343 (16.0%)	35/188 (18.6%)	
>10× ULN	197/343 (57.4%)	120/188 (63.8%)	
Inflammatory syndrome (n = 697), n/N (%)	437/459 (95.2%)	235/238 (98.7%)	**0.017**
IL-6 (n = 169), n/N (%)	43/97 (44.3%)	47/72 (65.3%)	**0.007**
NLR, Median (IQR) (n = 661)	9.1 (4.5; 16.8)	9.5 (5.7; 16.8)	0.178
PLR, Median (IQR) (n = 661)	318.6 (190; 464)	324.0 (185.1; 505.7)	0.527
**Imaging, n (%)**			
Chest CT lung involvement			**0.003**
<25%	153 (28.8%)	79 (27.7%)	
25–75%	233 (43.9%)	97 (34.0%)	
>75%	145 (27.3%)	109 (38.2%)	

Abbreviations: IQR, interquartile range; NLR, neutrophil-to-lymphocyte ratio; PLR, platelet-to-lymphocyte ratio; CRP, C-reactive protein; IL-6, interleukin-6; CT, computed tomography; ULN, upper limit of normal.

**Table 2:** Comparative Analysis of Clinical Course and Prognosis According to the Presence of Liver Injury at Admission.

Variable	Group 1 (No LI)	Group 2 (LI at Admission)	*p*-Value
**Length of hospitalization (days), Median (IQR)**	10.5 (7.0–15.0)	10.0 (6.0–15.0)	0.250
**ICU admission, n (%)**	192 (36.2)	126 (44.2)	**0.025**
**Need for mechanical ventilation, n (%)**			**0.011**
No	341 (64.2)	159 (55.8)	
CPAP/HFNO	65 (12.2)	38 (13.3)	
Invasive mechanical ventilation	125 (23.5)	88 (30.9)	
**Length of hospitalization in ICU (days), Median (IQR)**	6.0 (3.0–12.0)	5.0 (2.0–11.0)	0.141
**7-day mortality, n/N (%)**	74/459 (16.1)	66/255 (25.9)	**0.001**
**Mortality, n/N (%)**	167/454 (36.8)	126/253 (49.8)	**<0.001**

Abbreviations: IQR, interquartile range; ICU, intensive care unit; CPAP, continuous positive airway pressure; HFNO, high-flow nasal oxygen therapy.

**Table 3:** Comparative Analysis According to the Pattern of Liver Injury at Admission.

Variable	Cytolysis (N = 191)	Cholestasis (N = 39)	Mixed (N = 55)	*p*-Value
Median age (years (IQR))	70.0 (62.0–80.0)	70.0 (61.0–78.0)	64.0 (52.0–70.0)	**0.002**
**Gender, n (%)**				0.127
Male	91 (47.6)	17 (43.6)	34 (61.8)	
Female	100 (52.4)	22 (56.4)	21 (38.2)	
**Comorbidities, n (%)**				
Cardiovascular diseases	150 (78.5)	32 (82.1)	34 (61.8)	**0.024**
Pulmonary diseases	16 (8.4)	4 (10.3)	5 (9.1)	0.927
Malignancies	15 (7.9)	4 (10.3)	5 (9.1)	0.868
Diabetes mellitus	58 (30.4)	14 (35.9)	13 (23.6)	0.423
Obesity	45 (23.6)	9 (23.1)	12 (21.8)	0.964
Metabolic syndrome	24 (12.6)	6 (15.4)	5 (9.1)	0.643
**Clinical Symptoms, n (%)**				
Fever	99 (51.8)	19 (48.7)	31 (56.4)	0.748
Respiratory symptoms	174 (91.1)	36 (92.3)	47 (85.5)	0.413
Digestive symptoms	28 (14.7)	8 (20.5)	13 (23.6)	0.251
**Laboratory Parameters**				
NLR, Median (IQR)	9.5 (5.7–18.4) (n = 154)	10.9 (5.3–17.2) (n = 30)	9.3 (4.9–13.2) (n = 50)	0.576
PLR, Median (IQR)	351.0 (215.0–548.1) (n = 154)	298.4 (172.7–485.0) (n = 30)	263.3 (141.3–429.5) (n = 50)	0.050
Inflammatory syndrome, n/N (%)	152/152 (100.0)	32/34 (94.1)	51/52 (98.1)	**0.026**
**Chest CT Lung Involvement, n (%)**				0.319
<25%	50 (26.2)	10 (25.6)	19 (34.5)	
25–75%	63 (33.0)	18 (46.2)	16 (29.1)	
>75%	78 (40.8)	11 (28.2)	20 (36.4)	
**Clinical Outcomes, n (%)**				
ICU admission	85 (44.5)	18 (46.2)	23 (41.8)	0.908
Mechanical ventilation	84 (44.0)	18 (46.2)	24 (43.6)	0.965
Length of hospitalization (days), Median (IQR)	9.5 (5.0–14.0)	13.0 (7.5–22.0)	11.0 (7.0–15.0)	**0.035**
7-day mortality, n/N (%)	52/169 (30.8)	5/37 (13.5)	9/49 (18.4)	**0.039**
Overall in-hospital mortality, n/N (%)	89/168 (53.0)	16/36 (44.4)	21/49 (42.9)	0.361

Abbreviations: IQR, interquartile range; NLR, neutrophil-to-lymphocyte ratio; PLR, platelet-to-lymphocyte ratio; CT, computed tomography; ICU, intensive care unit. Continuous variables were compared using the Kruskal–Wallis test, with post-hoc Mann–Whitney U tests with Bonferroni correction when appropriate. Categorical variables were compared using the chi-square test or Fisher–Freeman–Halton exact test, as appropriate.

**Table 4:** Independent Predictors of Mortality and ICU Admission: Multivariable Logistic Regression Analysis.

Variable	Overall Mortality (n = 536) Adjusted OR (95% CI)	*p*	7-Day Mortality (n = 541) Adjusted OR (95% CI)	*p*	ICU Admission (n = 593) Adjusted OR (95% CI)	*p*
Age (years)	1.056 (1.035–1.078)	<0.001	1.035 (1.012–1.060)	0.003	0.999 (0.982–1.017)	0.942
Male sex vs. female	1.363 (0.880–2.109)	0.165	1.244 (0.761–2.034)	0.385	1.520 (0.995–2.321)	0.053
Cardiovascular comorbidities (present vs. absent)	0.876 (0.478–1.606)	0.668	0.960 (0.477–1.930)	0.908	0.957 (0.548–1.673)	0.879
Pulmonary comorbidities (present vs. absent)	1.068 (0.579–1.971)	0.833	1.038 (0.508–2.122)	0.919	0.918 (0.492–1.713)	0.788
Diabetes (present vs. absent)	1.267 (0.804–1.997)	0.308	1.522 (0.921–2.514)	0.101	1.176 (0.761–1.818)	0.466
Obesity (present vs. absent)	0.946 (0.541–1.655)	0.847	1.044 (0.545–1.999)	0.896	1.755 (1.025–3.002)	0.040
Inflammatory syndrome (present vs. absent)	2.329 (0.484–11.221)	0.292	Not reliably Estimable	—	1.862 (0.390–8.892)	0.436
NLR	1.040 (1.019–1.061)	<0.001	1.033 (1.016–1.050)	<0.001	1.056 (1.036–1.078)	<0.001
Liver injury at admission (present vs. absent)	1.757 (1.125–2.743)	0.013	1.824 (1.117–2.977)	0.016	0.988 (0.642–1.518)	0.955
Chest CT Lung Involvement						
25–75% vs. <25%	1.586 (0.899–2.799)	0.111	2.603 (1.169–5.796)	0.019	3.009 (1.674–5.411)	<0.001
>75% vs. <25%	10.646 (5.849–19.378)	<0.001	4.693 (2.147–10.257)	<0.001	16.539 (9.018–30.333)	<0.001

Abbreviations: OR, odds ratio; CI, confidence interval; ICU, intensive care unit; NLR, neutrophil-to-lymphocyte ratio; CT, computed tomography. Adjusted ORs and 95% CIs were obtained from multivariable binary logistic regression models. Reference categories were: absence of LI, female sex, absence of comorbidity, and <25% pulmonary involvement on chest CT. The effect of inflammatory syndrome on 7-day mortality could not be reliably estimated because of sparse outcome data/separation.

## Data Availability

The raw data supporting the conclusions of this article will be made available by the authors upon request.

## Abbreviations

ACE2	Angiotensin-Converting Enzyme 2
ADAMTS-13	A Disintegrin and Metalloproteinase with Thrombospondin Motifs 13
ALP	Alkaline Phosphatase
ALT	Alanine Aminotransferase
ARDS	Acute Respiratory Distress Syndrome
AST	Aspartate Aminotransferase
CI	Confidence Interval
CK	Creatine Kinase
CK-MB	Creatine Kinase-MB
COVID-19	Coronavirus Disease 2019
CPAP	Continuous Positive Airway Pressure
CRP	C-Reactive Protein
CT	Computed Tomography
GGT	Gamma-Glutamyl Transferase
HFNO	High-Flow Nasal Oxygen Therapy
ICU	Intensive Care Unit
IL-6	Interleukin-6
IMV	Invasive Mechanical Ventilation
IQR	Interquartile Range
LI	Liver Injury
MOF	Multiple Organ Failure
NIMV	Non-Invasive Mechanical Ventilation
NLR	Neutrophil-to-Lymphocyte Ratio
OR	Odds Ratio
PCR	Polymerase Chain Reaction
PLR	Platelet-to-Lymphocyte Ratio
SARS-CoV-2	Severe Acute Respiratory Syndrome Coronavirus 2
SD	Standard Deviation
SPSS	Statistical Package for the Social Sciences
TB	Total Bilirubin
ULN	Upper Limit of Normal
